# Design Concepts of Virus-Like Particle-Based HIV-1 Vaccines

**DOI:** 10.3389/fimmu.2020.573157

**Published:** 2020-09-30

**Authors:** Chun-Wei Chen, Narcís Saubi, Joan Joseph-Munné

**Affiliations:** ^1^Microbiology Department, Vall d'Hebron Research Institute (VHIR), Barcelona, Spain; ^2^EAVI2020 European AIDS Vaccine Initiative H2020 Research Programme, London, United Kingdom; ^3^Microbiology Department, Hospital Universitari de la Vall d'Hebron, Barcelona, Spain

**Keywords:** HIV-1, vaccine, virus-like particles, broadly neutralizing antibodies, cytotoxic T- lymphocyte response

## Abstract

Prophylactic vaccines remain the best approach for controlling the human immunodeficiency virus-1 (HIV-1) transmission. Despite the limited efficacy of the RV144 trial in Thailand, there is still no vaccine candidate that has been proven successful. Consequently, great efforts have been made to improve HIV-1 antigens design and discover delivery platforms for optimal immune elicitation. Owing to immunogenic, structural, and functional diversity, virus-like particles (VLPs) could act as efficient vaccine carriers to display HIV-1 immunogens and provide a variety of HIV-1 vaccine development strategies as well as prime-boost regimes. Here, we describe VLP-based HIV-1 vaccine candidates that have been enrolled in HIV-1 clinical trials and summarize current advances and challenges according to preclinical results obtained from five distinct strategies. This mini-review provides multiple perspectives to help in developing new generations of VLP-based HIV-1 vaccine candidates with better capacity to elicit specific anti-HIV immune responses.

## Introduction

Human immunodeficiency virus-1 (HIV-1), which causes acquired immunodeficiency syndrome (AIDS), was discovered in the early 1980s, and since then it has become a global epidemic. At the end of 2018, ~37.9 million people were living with HIV and 1.7 million people became newly infected. Among HIV-infected individuals, 36.2 million (96%) were adults and 1.7 million (4%) were children. The pandemic of HIV differs considerably between regions and countries. According to WHO, Africa is the most severely affected region, with almost 1 in 25 adults (4%) infected with HIV and accounting for roughly 2 in 3 (66%) of the HIV-1 patients worldwide (1). The development of antiretroviral therapies (ART) has significantly reduced morbidity and mortality associated with HIV-1 infection worldwide (2); nevertheless, it might lose efficacy due to HIV-1 resistance (3). Although ART can achieve control of viral load to an undetectable level, it fails to thoroughly clear HIV-1 because ART only acts upon activated replicating viruses rather than the latent reservoirs (4). The exposure of HAART is likely to be life-long due to the chronic HIV infection. Additionally, long-term side effects are commonly reported in HIV-1 patients under ART treatment, which may conceivably become even more frequent with the increasing age (5). Pre-exposure prophylaxis (PrEP) is currently the most effective preventive approach against HIV-1 infection (108). However, the PrEP program is unaffordable for many highest HIV-1 prevalence countries. Also, using PrEP may cause significant adverse effects. For example, Truvada has been proven to affect bone density and kidney functions (6). Therefore, developing an affordable, efficacious and safe HIV-1 prophylactic vaccine is the most needed strategy for ultimate control of the HIV-1 epidemic. Since the first HIV-1 vaccine clinical trial took place in 1987 (7), a series of vaccine candidates with different strategies have been tested in more than 230 Phase I/II/III clinical trials in both developed and developing countries (8). The landmark HIV-1 vaccine trial (RV144) in Thailand that used a heterologous combination with a canarypox virus vector (ALVAC/HIV) expressing Gag, Pol, and gp120 as a prime and a bivalent gp120 protein boost revealed a modest efficacy of 31.2% against HIV-1 acquisition (9). Extensive post-trial studies defined the immune correlates of vaccine protection in the RV144 trial and identified a set of immunological end points, such as anti-V1-V2 antibodies, IgG3, or IgA antibodies. No significant neutralizing antibodies or cell-mediated immunity were detected (10). This progress indicated that structural-based vaccine design for inducing antibodies against HIV-1 could be feasible if investigators can overcome challenges of searching efficient nanoparticles as a vaccine carrier to stabilize HIV-1 antigens for optimal immune elicitation.

## Basic Concepts of Virus-Like Particle-Based Vaccines

Virus-like particles (VLPs) have several advantages over other traditional vaccine strategies. VLPs are self-assembling, non-infectious, and structurally authentic virions that are able to conformationally display antigens on its surface and contribute to more robust humoral and cell-mediated immunity against viral infection (11–14). In particular, these distinctive features of VLPs make them immunologically omnipotent, structurally diverse, and functionally versatile (15). VLPs could be utilized as delivery agents without the help of adjuvants for a wide range of vaccine candidates. In this review, we will discuss (I) immunogenic, structural, and functional aspects of VLPs that offer a variety of HIV-1 vaccine development strategies; (II) clinical progress of the VLP-based HIV-1 vaccines and (III) the current advances and challenges of VLP-based HIV-1 vaccines.

### Immunogenicity of VLPs

VLPs are stimulators of innate immunity. Innate immune recognition against viral infection is controlled by the pattern recognition receptors, such as Toll-like receptors (TLRs), in the cytosol of infected cells or on the cell surface. TLRs recognize viral proteins and genome through the pathogen-associated molecular patterns (PAMPs) (16, 17) and activate antigen-presenting cells (APCs), which stimulate downstream T and B cell immunity.

The most effective T cell-mediated immunity is elicited by either the viral vector used alone or as a booster after DNA priming, because they result in endogenous expression of viral proteins by transduced cells. Owing to the unique structural features, VLPs can be efficiently taken up by dendritic cells (DCs) through endocytic processes. The DCs subsequently undergo maturation and induce cellular immune responses, such as cytokine production and CD4+ T-helper cell activation, through MHC class II pathway (18). Furthermore, compared with other exogenous immunogens, VLPs can also trigger MHC class I pathway in the absence of viral infection (18) and further stimulate CD8+ cytotoxic T-lymphocyte (CTL) responses (19, 20).

VLPs have predominantly been used to induce humoral immunity (21). Antigens presented on the repetitive structures of VLPs contribute to enhancement of cross-linking with B cell receptors (22) and drive the B cell's somatic hypermutation as well as immunoglobulin class switching from the IgM to the IgG (23). VLPs could promote B cell differentiation to plasma cells, which secrete IgG2a class-switched antibody (24). VLPs are also able to trigger TLR-mediated B cell activation and increase overall IgG levels (25). The efficient production of long-lived B cells offers an explanation for the high potency of VLP-based vaccines even when administered in one dose without boosting (26).

### Structural Diversity of VLPs

**Figure 1:**
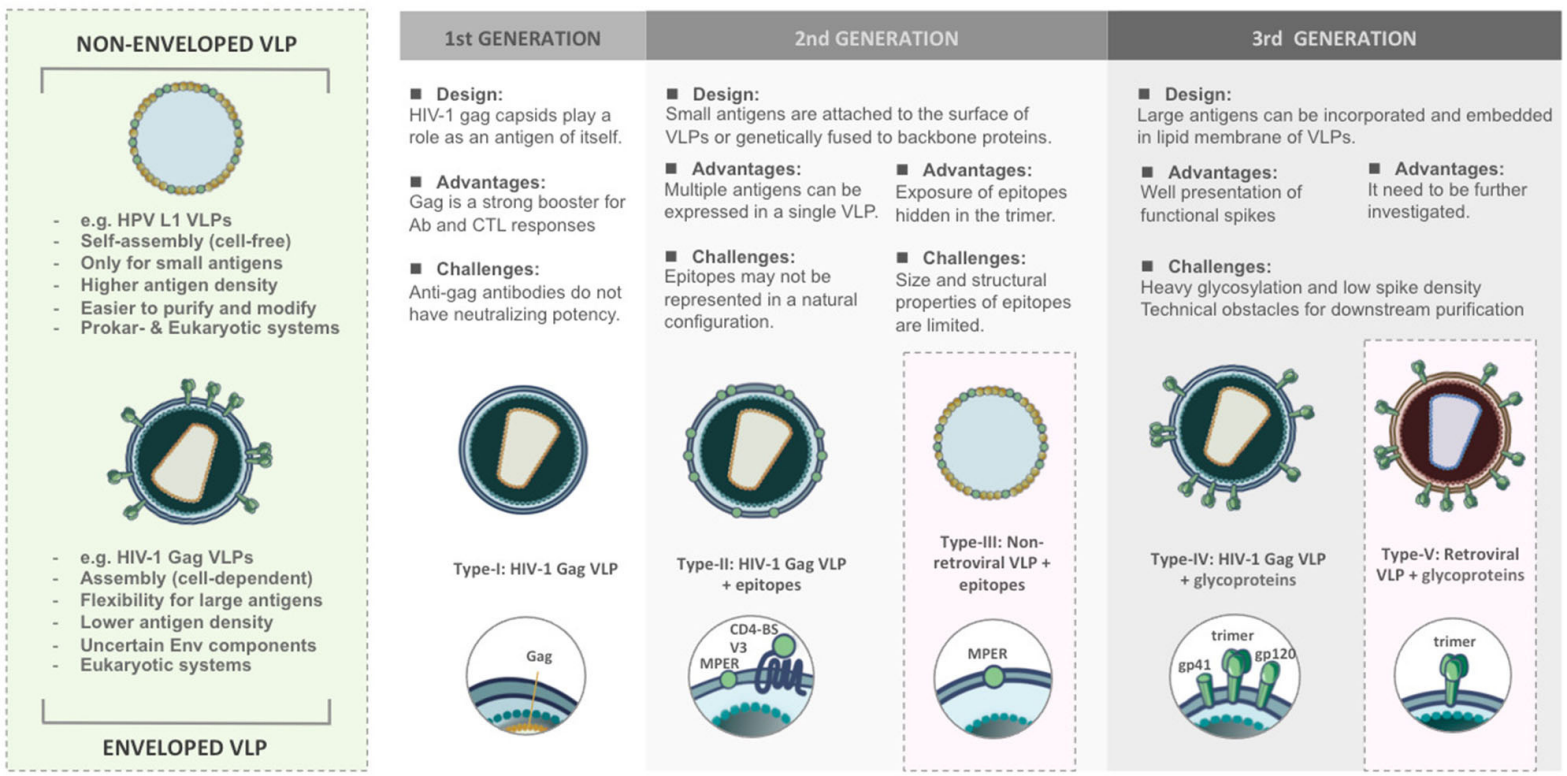
Comparison of structural features and functional versatility of VLPs corresponding to VLP-based HIV-1 vaccine design.

According to structural features, VLPs are classified into non-enveloped and enveloped VLPs (Figure 1). Non-enveloped VLPs (non-eVLPs) can be constructed from single or multiple capsid proteins without the cell membranes. Structurally simple non-eVLPs, such as human papillomavirus (HPV) L1 VLPs, can be synthesized by using eukaryotic (27) or prokaryotic expression systems (28) and self-assemble into single-capsid VLPs in a totally cell-free condition (29). By contrast, multiple-capsid non-eVLPs are more complicated and technically challenging (30). For example, HPV L1-L2 VLPs are only generated in eukaryotic systems, which are capable of co-expressing two different capsids and forming VLPs within a cell environment (31).

In contrast to non-enveloped VLPs, enveloped VLPs (eVLPs), such as HIV-1 eVLPs, can only be produced by eukaryotic systems. Undoubtedly, the mammalian cell systems have the most precise and complex post-translational modification that is optimal for constructing eVLPs (32). The viral envelopes (Env) typically include cell membranes derived from host cells during budding and glycoproteins embedded in the lipid bilayers (33, 34). The cell-derived membranes provide additional flexibility to integrate heterologous antigens and adjuvants. However, this flexibility increases the risk of containing the uncertain host cellular components in eVLPs which may affect downstream purification processes and raises technical challenges as well as obstacles for regulatory approval (35). eVLPs are rarely characterized biophysically because their structures are less uniform. In different virus families, the composition of viral Env changes and usually depends on the assembly process as well as cell strains used for production (36).

### Functional Versatility of VLPs

The versatility of VLPs brings with it different patterns in presenting immunogens and contributes to a wide range of applications as HIV-1 vaccine platforms (Figure 1).

The first generation of VLPs takes on itself as an immunogen, such as most of the licensed VLP-based vaccines. For instance, Cervarix® and Gardasil® are VLP-based vaccines against HPV infection. HPV L1 capsids could spontaneously assemble into 60 nm non-eVLPs and induce neutralizing antibodies (37). In the case of HIV-1, Assembly and release of HIV-1 precursor Pr55/Gag VLPs from recombinant baculovirus expression systems could strongly trigger cellular responses and antibody production even though such antibodies do not have neutralizing potency (38, 39).

The second generation of VLPs was developed as a result of presenting epitopes on the surface of VLPs either by genetic fusion or chemical conjugation (40). The chimeric VLPs provide a platform to induce antibodies targeting defined epitopes against HIV-1 (41) and also various diseases (42, 43). However, genetic and chemical techniques have their limitation. Genetically modified capsids might fail to build up the complete VLPs, in particular, if the antigens are too big to be displayed. Conjugating epitopes on VLPs is difficult to achieve the natural conformation and structural authenticity to those found on the native virions. It suggests that conformational integrity is critical for the immunogenicity of VLPs.

The third generation of the VLPs can be defined as expressing large antigens, such as HIV-1 functional spikes, on eVLPs. HIV-1 glycoproteins act as principal immunogens to trigger broadly neutralizing antibodies (bnAbs) (44). Due to complexity around maintaining the structural authenticity of bnAb epitopes, glycoproteins require being properly incorporated and embedded in the lipid membrane. This concept has been demonstrated in most of the HIV-1 Gag VLPs (45, 46). However, the design of eVLPs needs more effort to meet purification challenges and overcome unstable Env composition.

## VLP-Based HIV-1 Vaccine Candidates in the Clinical Trials

Regarding VLP-based HIV-1 vaccine candidates, only a few prototypes have been assessed in clinical trials. The first VLP-based HIV-1 vaccine candidate in phases I/II studies was the therapeutic HIV-1 p24-VLP derived from Gag capsid. Vaccination with the p24-VLP had been demonstrated to be safe, and no serious adverse events were detected in healthy volunteers (47). Nonetheless, the p24-VLP vaccine was poorly immunogenic, and did not significantly increase the humoral and cellular immune responses (48, 49). Despite the speculation that the development of enveloped VLP-based vaccines might face some technical challenges, the standstill of VLP-based HIV-1 vaccines in clinical trials could be attributed to the failure of showing efficacy in pre-clinical non-human primates (NHPs) challenge models. The use of an SIV model of the human vaccine is very questionable, especially for Env-based vaccines, because the gp120 Env between SIV and HIV is significantly different (50). Additionally, the putative immunoglobulin germline predecessors of highly mutated bnAbs are distinct between human and rhesus macaques (51).

## Advances and Challenges of VLP-Based HIV-1 Vaccine Development

The designs of VLP-based HIV-1 vaccines has evolved from 1st generation of capsid-oriented to 2nd generation of epitope-focused to 3rd generation of envelope-based vaccines that include native forms of Env trimers and sequential Env antigens predicted to elicit bnAbs. Simultaneously, various VLP-based vaccine platforms have been tested to enhance the immunogenicity of HIV-1 antigens. According to different construction strategies, VLP-based HIV-1 vaccines can be categorized into five types (Figure 1). We briefly review the current progress and challenges in the development of VLPs as a vaccination approach against HIV-1.

### Type-I: HIV-1 Enveloped VLPs Acting as Homologous Immunogens

The early strategy of HIV-I VLP vaccine construction is based on viral capsid (Figure 1). Without the participation of gp120 Env which is quite different between human and NHPs model, the vaccine candidates would be easier to pass SIV challenge. HIV-1 Gag capsid protein is a major component of 100–120 nm HIV-1 VLPs, which is capable of assembling and budding from the cell membrane (38). Gag acts as an effective booster for Gag-specific cellular and humoral immunity, especially CTL responses, in mouse (39, 52), and rhesus macaque models (53). However, it has been demonstrated that such antibodies were not involved in neutralizing activities in humans (49). The immunity elicited by Gag VLPs mainly depends on the structure, and this finding could be considered in the design of HIV-1 vaccines (54).

### Type-II: HIV-1 Enveloped VLPs Expressing HIV-1 Epitopes

The type-II/III HIV-1 VLP design strategy is epitope-focused and could be applied on both eVLP and non-eVLP (Figure 1). Over the past decades, many bnAbs and bnAb epitopes on the HIV-1 Env have been identified. They are mainly located at CD4-binding site (CD4-BS), membrane proximal region (MPER), high mannose patch (V3 region), the Env trimer apex (V1/V2 region) and gp120/gp41 interface region. The early attempts at Gag VLP-based HIV-1 vaccines heavily relied on genetic fusion techniques. Unfortunately, without a clear concept of the structural integrity of epitopes, humoral immunity elicited by inserted antigens were relatively weak. In attempts to increase the immunogenicity of recombinant antigens, Gag VLPs could also play a role as a platform for carrying HIV-1 epitopes, glycoproteins, or even Env trimers. Several preclinical studies found that assembly and extracellular release of Gag VLPs were not influenced by coupling with HIV-1 epitopes, monomeric gp120 (55) or even trimeric gp140 spikes (56). For instance, in many immunization studies, Gag-eVLPs fused with variable V3 loop epitopes (57, 58), CD4 binding domains of gp120 (58) or MPER of gp41 (59) only achieved low antibody responses and were incapable of HIV-1 neutralization. Exceptionally, a few studies pointed out that V3 immunodominant domains expressed on Gag VLPs did not influence DCs presenting exogenous antigens in MHC class I-mediated manner. Therefore, CTL responses against V3 loops could be distinctly detected in immunized BALB/c mice (57, 58).

### Type-III: Non-enveloped VLPs Fusing With HIV-1 Epitopes

Direct exposure of the HIV-1 epitopes that are hidden in Env trimer might be a feasible strategy to induce nAbs and CTL responses. The structurally simple non-eVLPs, which have advantages of easier construction and purification, offer a vehicle to implement this concept. A previous study revealed that highly conserved membrane proximal region (MPER) of HIV-1 Env expressed on the surface of bovine papillomavirus (BPV) L1 VLPs induced 2F5 and 4E10-specific nAbs in mice and resulted in a cross-clade neutralization. Nevertheless, direct presentation of 2F5 and 4E10 epitopes on BPV VLPs cannot achieve nAb production (41). In another study, the C-terminal alpha-helix of gp41 MPER expressed on bacteriophage-based VLPs have been demonstrated to develop cross-strain nAbs (60). These indicate that epitope-based vaccine approaches for priming nAbs heavily depend on the structural properties of neutralizing epitopes. The linear epitopes derived from MPER might be easier to maintain its structural authenticity on the VLPs (61). On the other hand, it has been demonstrated that vaccine design on the basis of BPV L1 VLPs carrying P18I10 CTL epitopes from HIV-1 V3 loops can elicit a strong cell-mediated immunity (62).

### Type-IV: HIV-1 Enveloped VLPs Presenting HIV-1 ENV

The field of VLP-based HIV-1 vaccines has recently shifted toward type-IV/V Env-based designs that include “native” forms of Env trimers and sequential Env immunogens to induce bnAbs against diverse circulating strains (Figure 1). HIV-1 Env spike is synthesized as a gp160 precursor and processed by viral protease into a heterodimer including three gp120 and three gp41 subunits (63). HIV-1 Gag VLP expressing un-cleaved gp160 (64), monomeric gp120 (65), trimeric gp140/gp41 (56), and whole Env trimer (66–69) have been tested in several animal models. In most of the trials, the elicitation of Env-specific antibody responses and cross-clade neutralization potencies were detected. Moreover, a few studies also found significant CTL responses targeting V3 loop regions (65, 68). From these results, Env trimers have been believed to be the primary antigens for VLP-based HIV-1 vaccine design, and a great deal of efforts has been made to improve its performance for priming bnAbs. However, the potency and breadth of neutralization are strongly inhibited by the high degree of genetic sequence variability amongst HIV isolates and the poor accessibility to the bnAb epitopes due to particular features of native Env trimers described below.

Heavy glycosylation forms a glycan shield on the surface of the HIV-1 Env spike, which covers the bnAb epitopes and reduces neutralization sensitivity (70, 71). For example, the CD4-binding site (CD4-BS) is highly conserved and buried in the Env trimer. HIV-1 evolves a heavy glycan shield around CD4-BS to hinder the bnAbs development (72). The deletion of the glycosylation site helped Env bind B cell receptors expressing two potential bnAbs, VRC01, and NIH45-46 (73). In another study, the deficiency of shielding glycan led to the exposure of quaternary neutralizing epitopes to CD4-BS and enabled the development of broad cross-neutralizing antibodies (74). De-glycosylation can be a feasible strategy to facilitate the exposure of bnAb epitopes in Env trimer and reinforce the potency of HIV-1 vaccines.

The density of HIV-1 Env spike (7–14 spikes/virion) is much lower than other viruses, even compared to the related SIV (~70 spikes/virion) (75). HIV-1 evolves a defense mechanism of presenting low density of Env spikes to prohibit antibody bivalent binding and further decrease avidity and impede neutralization (76). The density of Env spikes is important for an effective B cell receptor (BCR) cross-linking which contributes to B cell expansion, antibody affinity maturation, and bnAb production (77). Therefore, the previous studies indicated that the substitution of the transmembrane domain of gp41 by a heterologous, Epstein-Barr virus gp220/350-derived membrane anchor led to effective incorporation of gp120 to Gag VLPs (78). Similarly, replacement of transmembrane (TM) regions, signal peptide, and cytoplasmic tail domains of HIV-I Env glycoproteins to other viral or cellular functional peptides, respectively, can improve Env spike incorporation (79). Genetic modification of TM regions of HIV-1 Env spikes seems to be a practical approach to boost nAb responses.

### Type-V: Retroviral Enveloped VLPs Presenting HIV-1 ENV

Chimeric simian immunodeficiency virus (SIV) Gag VLPs, presenting modified HIV-I Env glycoproteins with de-glycosylation and V1/V2 loop deletion, have been demonstrated to induce cellular and humoral immunity with neutralizing activities against HIV-1 (80). However, the mechanism and practical application of these chimeric SHIV eVLPs still need to be further investigated and explored.

## Prime-Boost Regimes of VLP-Based HIV-1 Vaccines

**Figure 2:**
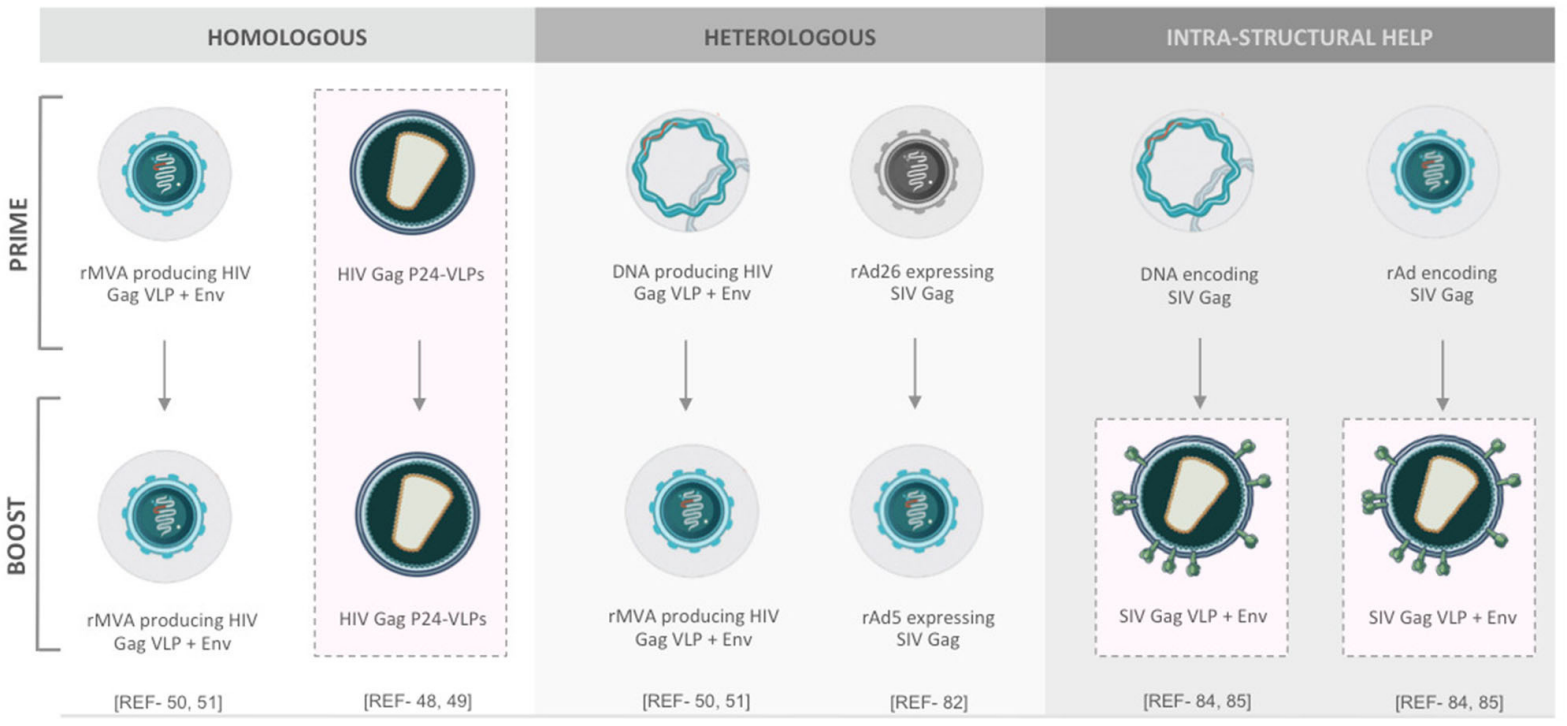
Different prime-boost regimens for VLP-related HIV-1 vaccines.

The gap between host immunity and immune correlates of vaccine protection against HIV-1 is not comprehensively defined (10). Over the past decade, many different prime-boost formats of VLP-based HIV-1 vaccine have been tested (Figure 2). Recently, a few studies have pointed out a viewpoint that heterologous prime-boost regimens, may contribute to more augmented immunogenicity (81). To endorse these concepts, a heterologous prime-boost regimen of T cell-based vaccines, which is done with two serologically distinct adenovirus vectors expressing the same SIV Gag as immunogens, could elicit more robust CTL responses compared with the homologous regimen following the SIV challenge of rhesus monkey models (82). In another study, the state-of-the-art synergistic effects between lentiviral Env and other non-Env proteins on VLPs may also lead to stronger immunogenicity. A significantly high level of Env-specific Ab responses was detected in mice immunized with adenovirus vectors (or DNA vaccines) encoding SIV Gag-Pol and subsequently boosted with SIV eVLPs containing Gag-Pol and Env glycoprotein (83). The synergistic effect, also known as intra-structural help, between Gag-Pol-specific CD4 T helper cells and Env-specific B cells provides a possible explanation (84). All of these results hint toward the fact that thinking outside the box is needed for HIV-1 vaccine design, formulation, and regimen in the future.

## Expert Commentary

In spite of such encouraging proof-of-concept studies, very few of these VLP-based HIV-1 vaccine candidates have proceeded to clinical trials over past two decades. Some of the reasons for the stagnation of VLP-based HIV-1 vaccines in human clinical trials could be (i) the failure of showing efficacy in pre-clinical rhesus macaques challenge models. Because the Env gp120 is significantly different between HIV-1 and SIV, it results in the use of an SIV version of the human HIV-1 vaccine is highly debatable; (ii) the technical and regulatory obstacle. In particular, the uncertain components in VLPs may affect downstream purification processes, end-point immunogenicity, and even toxicity. Production and purification development of VLP-based HIV-1 vaccines still rely heavily on substantial expertise and knowledge gained from industrial experiences; (iii) Another difficulty for the design of VLP-based HIV-1 vaccines is to ensure the epitopes presented on VLPs achieve the greatest conformational authenticity to those found on the native Env trimers. Some bioinformatic techniques, such as SWISS homology modeling, might be helpful to predict HIV-1 epitope presentation on VLPs. The lessons learned from the past failed trials may inspire us to more rational vaccine design. However, human immune systems are complicated and still have many unknowns. New prospects and out-of-the-box thinking might be needed for the future VLP-based HIV-1 vaccine development.

## Author Contributions

C-WC wrote the manuscript and designed the figures. NS provided intellectual consultation and revision. JJ-M edited and revised the overall mini-review manuscript. All authors agreed to be accountable for the content of the work. All authors contributed to the article and approved the submitted version.

## Conflict of Interest

The authors declare that the research was conducted in the absence of any commercial or financial relationships that could be construed as a potential conflict of interest.
